# Case reports of subcutaneous pdC1INH in pregnancy and lactation: expanding treatment options for hereditary angioedema in Portugal

**DOI:** 10.3389/falgy.2025.1604440

**Published:** 2025-07-02

**Authors:** Ana Luísa Pinhal, Natacha Santos, Eunice Dias de Castro

**Affiliations:** ^1^ Department of Allergy and Clinical Immunology, Unidade Local de Saúde de São João, Porto, Portugal; ^2^ Department of Immunoallergology, Unidade Local de Saúde do Algarve, Faro, Portugal; ^3^ Department of Public Health and Forensic Sciences and Medical Education, Faculty of Medicine, University of Porto, Porto, Portugal

**Keywords:** hereditary angioedema, lactation, long-term prophylaxis, plasma-derived C1 inhibitor, pregnancy

## Abstract

Hereditary angioedema (HAE) is a rare genetic disorder characterized by recurrent episodes of subcutaneous and/or submucosal angioedema. Pregnancy and breastfeeding may be associated with an increased frequency of attacks. Plasma-derived C1 inhibitor (pdC1INH) is the recommended first-line treatment for long-term prophylaxis (LTP) in these special populations. The pdC1INH currently available in Portugal is one intravenous (IV) formulation not approved for LTP, as are the other IV and subcutaneous (SC) formulations. This report documents the first cases of SC pdC1INH use during pregnancy and breastfeeding in Portugal. It describes two cases of 37-year-old women with HAE type 1 treated with SC pdC1INH as LTP during pregnancy and lactation. Both patients had been previously treated with tranexamic acid. In the first case, the patient was started on IV pdC1INH at 8 weeks' gestation due to clinical deterioration. Due to difficult IV access and inability to space out administrations, SC pdC1INH at a dose of 4,000 U (∼43.5 U/kg) every 72 h was started at 21 weeks' gestation. Administration intervals were progressively increased to 96 and later 120 h. LTP was continued throughout lactation. In the second case, LTP was not administered during pregnancy. However, after delivery, the patient experienced a worsening of angioedema episodes during breastfeeding, which persisted despite tranexamic acid treatment. SC pdC1INH was started six months postpartum at a dose of 2,000 U (∼45 U/kg) twice weekly. The administration interval was later increased to 120 h. Both patients remained free of angioedema episodes and reported no systemic adverse events. The safety of SC pdC1INH was consistent with reports in the literature. Overall, these positive results support the future use of SC pdC1INH in a broader population of pregnant and lactating women in clinical practice.

## Introduction

Hereditary angioedema (HAE) is a rare genetic disorder characterized by recurrent episodes of localized cutaneous and submucosal angioedema (1). The most commonly affected areas include the skin (especially the extremities, face, and genitals) and the gastrointestinal tract. Other sites may be affected, and laryngeal attacks are of particular concern due to their potential to cause fatal airway obstruction (1, 2). The recurrent, unpredictable and potentially life-threatening nature of HAE attacks has a significant impact on patients' quality of life (3), resulting in physical, emotional, and psychological distress.

HAE can be classified into HAE due to C1 inhibitor (C1INH) deficiency (HAE-C1INH) and HAE with normal C1 inhibitor (HAE-nC1INH). Patients with HAE-C1INH may have low plasma levels of C1INH protein (HAE type 1) or normal to elevated plasma levels of dysfunctional C1INH protein (HAE type 2) (4).

Regarding treatment, the primary goals in HAE are to achieve complete disease control and enable patients to lead normal, unrestricted lives (1, 5). Often, this can only be achieved with the use of long-term prophylaxis (LTP). In Portugal, the following drugs are commercially available for LTP: lanadelumab and berotralstat as first-line therapies; danazol and antifibrinolytics (e.g., tranexamic acid), which are classified as second-line therapies according to international guidelines; and an intravenous (IV) plasma-derived C1INH (pdC1INH), which is only approved for on-demand treatment and short-term prophylaxis (STP) (1, 6).

Pregnancy may increase, decrease, or have no effect on the frequency and severity of HAE attacks (1). However, an increase in disease activity during pregnancy appears to be the most common scenario (7, 8). Notably, the course of HAE in a previous pregnancy does not reliably predict how the disease will evolve in subsequent pregnancies (7). The course of the disease during gestation is also unpredictable, with some studies reporting a worsening of angioedema attacks during the first trimester, and others reporting a higher frequency of attacks in the last trimesters (9–11).

During eutocic delivery, the risk of experiencing an angioedema attack is low, and STP is not routinely recommended (12). However, STP is recommended in cases of dystocic delivery, such as cesarean sections or instrumented vaginal deliveries (7).

Breastfeeding may be associated with an increased frequency of angioedema attacks in women with HAE, possibly related to elevated serum prolactin levels (10).

During pregnancy and lactation, therapeutic options for the management of HAE are limited. Attenuated androgens are absolutely contraindicated during pregnancy as they may lead to virilization of the female fetus (13). In addition, danazol should also be avoided during lactation, as it can potentially decrease milk production (14). The safety of lanadelumab and berotralstat during pregnancy and lactation has not been established (1, 15). Antifibrinolytics may be used during pregnancy despite lack of proven efficacy and potential increase in thrombotic risk (1, 7). Tranexamic acid appears to be safe during lactation (16). PdC1INH has been used during pregnancy and lactation with proven safety and efficacy and is the recommended LTP in these special populations (1).

In Portugal, only IV pdC1INH is currently available for on-demand prophylaxis and STP. Subcutaneous (SC) pdC1INH has not been approved for commercialization by INFARMED and can only be used by special authorization.

The aim of this report was to describe the first cases of women with HAE treated with SC pdC1INH for LTP during pregnancy and lactation in Portugal.

## Case presentation

This report describes the cases of two women with HAE followed at the Department of Allergy and Clinical Immunology of two Portuguese centers, who were treated with SC pdC1INH during pregnancy and lactation. The first patient started treatment in March 2024 during pregnancy, and the second patient started treatment in June 2024 during lactation.

## Case 1

### Patient information, clinical findings, and diagnostic assessment

The first case is a 37-year-old woman with HAE type 1 (Table 1). Her diagnosis was established at the age of 20 by C4 and C1INH measurement and later confirmed by genetic testing, which identified the p.Ala275Thr variant in exon 5 of the SERPING1 gene.

**Table 1:** Summary of treatment timeline and outcomes.

Cases	Age (in years)	SC pdC1INH initiation date	SC pdC1INH initiation dose	SC pdC1INH dose adjustments	Adverse events	Outcomes
Case 1	37	21st week of gestation	43.5 U/kg every 72 h	Dosing intervals increased to 96 h at the 23rd week of gestation and to 120 h at the 29th week of gestation.	Mild pain at the injection site	No recurrence of angioedema attacks following treatment initiation
Case 2	37	6 months postpartum	45 U/kg twice weekly	Dosing intervals increased to 120 h at 8 months postpartum	Mild pain at the injection site	No recurrence of angioedema attacks following treatment initiation

At the age of 19, shortly after starting an estrogen-containing contraceptive pill, the woman experienced her first angioedema attack, which affected the abdomen and feet. During her first pregnancy at the age of 32, she experienced a moderate worsening of the disease, especially during the first trimester, with weekly abdominal HAE attacks. She was treated with on-demand IV pdC1INH, but declined the proposed option of LTP with the same drug.

At the age of 36, she was on LTP with tranexamic acid (1,000–1,500 mg/day) with only partial HAE control. She experienced angioedema attacks approximately every 2 months, primarily peripheral and typically induced by stress. This treatment was discontinued when she decided to become pregnant. After the first few weeks of pregnancy, there was a marked increase in HAE attacks, which significantly affected the patient's quality of life. In the 8th week of pregnancy, a joint decision was made to start IV pdC1INH at a dose of 1,500 U (∼16 U/kg) twice a week.

At 15 weeks' gestation, as the patient remained attack-free but had difficult IV access, the frequency of IV pdC1INH administration was reduced to once a week. However, due to the recurrence of HAE attacks, a special authorization for the use of SC pdC1INH was requested from INFARMED.

### Therapeutic intervention and outcome

After INFARMED's approval, the patient started LTP with SC pdC1INH at a dose of 4,000 U (∼43.5 U/kg) every 72 h at 21 weeks' gestation. After three hospital administrations and patient and family education, the treatment was successfully transitioned to home administration.

At 24 weeks' gestation, the administration interval was increased to 96 h, and at 29 weeks' gestation, it was further increased to every 120 h (i.e., every five days). The patient remained attack free throughout this period and reported no side effects other than mild pain at the injection site.

A healthy baby boy was delivered at 38 weeks gestation. STP with IV pdC1INH 1,500 U was administered prior to cesarean section and the delivery was uneventful. After delivery and throughout lactation, LTP was continued with SC pdC1INH 4,000 U (∼43.5 U/kg) every five days. The patient remained free of angioedema attacks and with good disease control, as evidenced by an Angioedema Control Test (AECT) score of 15, an Angioedema Quality of Life Questionnaire (AE-QoL) score of 20, and an Angioedema Activity Score (AAS) of zero at five weeks postpartum, all collected in paper forms during medical appointments, as part of the routine clinical assessment.

## Case 2

### Patient information, clinical findings, and diagnostic assessment

The second case is another 37-year-old woman with HAE type 1 (Table 1). She experienced her first episode of angioedema at the age of 15 following a dental procedure, with swelling predominantly in the limbs and abdomen. The diagnosis was established at the age of 32 through the detection of C1-INH protein deficiency. Genetic testing further identified the SERPING1 c.1480C > T mutation. The patient was started on LTP with tranexamic acid, which resulted in a reduction in the frequency and severity of angioedema attacks.

During her first pregnancy at the age of 32, the disease remained under control without the need for LTP and delivery was uneventful. However, during breastfeeding, the frequency of angioedema attacks increased and LTP with tranexamic acid was initiated with a good clinical response.

During her second pregnancy, at the age of 36, the disease was managed exclusively with on-demand treatment. In the second trimester, the patient experienced an increase in the frequency of episodes, with abdominal angioedema occurring once or twice a week. These episodes resolved with medical leave and rest. Delivery occurred at 38 weeks' gestation under STP with IV pdC1INH and was uneventful.

During breastfeeding, the patient experienced an increase in the frequency of abdominal and peripheral angioedema attacks, prompting the initiation of LTP with tranexamic acid at a dose of 2,000 mg daily. Despite prophylaxis, she continued to experience weekly angioedema episodes lasting an average of two days each, with a significant impact on her quality of life. Special authorization for the use of SC pdC1-INH was requested and granted.

### Therapeutic intervention and outcome

At six months postpartum, treatment with SC pdC1INH was initiated at a dose of 2,000 U (∼45 U/kg) administered twice weekly. After five in-hospital administrations and training, the patient transitioned to home administration. The only adverse event reported was tolerable pain in the injection site.

Following the introduction of SC pdC1INH LTP, the patient remained free of angioedema attacks. Two months later, based on the good clinical response as evidenced by an AECT score of 16, an AE-QoL score of zero, and an AAS of zero, combined with a reduction in breastfeeding frequency, the SC pdC1INH administration interval was increased to every 120 h (i.e., every five days).

Currently, both women continue to breastfeed, with treatment duration adjusted according to clinical progression.

## Discussion

The efficacy and safety of SC pdC1INH as LTP at the doses of 40 and 60 U/kg administered twice weekly was previously demonstrated in the COMPACT (Clinical Study for Optimal Management of Preventing Angioedema with Low-Volume Subcutaneous C1-Inhibitor Replacement Therapy) open-label extension study (17). In this study, four women were exposed to SC pdC1INH during the first pregnancy trimester without complications, and all delivered healthy babies (17). Additionally, a few published case reports have further confirmed the effectiveness and safety of SC pdC1INH as HAE prophylaxis during pregnancy and breastfeeding (18, 19).

In this report, the first patient experienced an increase in angioedema attacks during the first pregnancy trimester. Prophylaxis with IV pdC1INH successfully achieved complete disease control. However, the need for twice-weekly hospital visits and the difficulty in IV access hampered treatment adherence. It should be noted that several IV pdC1INH preparations are available, but Berinert^©^ is not approved for LTP (6) and Cinryze^©^, although approved for LTP, is not available in Portugal (20).

The second patient experienced an increase in the frequency of angioedema attacks during the second pregnancy trimester, but the symptoms were controlled with rest. However, during breastfeeding, she experienced weekly angioedema attacks before the introduction of SC pdC1INH.

The administered doses, 43.5 UI/kg in the first case and 45 UI/KG in the second, did not exactly match the recommended dosage of 60 UI/kg due to limitations related to the fixed-dose format of the syringes used for subcutaneous administration (1).

In both cases, stopping breastfeeding itself may have reduced the number of angioedema episodes in the postpartum period (7). Nevertheless, breastfeeding is widely recommended due to its significant benefits for the newborn (1).

Since the initiation of SC pdC1INH, both patients have remained completely asymptomatic, consistent with findings from a similar case report (18). The complete clinical response allowed the administration interval to be extended to every five days while maintaining effective symptom control. Apart from mild injection site pain, no relevant side effects were reported.

Although SC pdC1INH has been shown to be effective and safe during pregnancy and lactation, its use in Portugal is limited by the requirement to obtain it from abroad through a special authorization, resulting in delays in access to this life-changing treatment.

To the authors' knowledge, these are the first two cases of SC pdC1INH use in Portugal. The authors emphasize the importance of individualized treatment and highlight the potential of SC pdC1INH as a therapeutic option for other pregnant and lactating women in the country.

## Patient perspective

Case 1: “When I started using subcutaneous Berinert®, the changes in my life were significant: I gained more independence (no need to go to the hospital for intravenous medication) and more security (my disease is under control with effective medication and no side effects). I feel that my life has returned to normal, which has had a very positive impact on both my physical and mental well-being.”

Case 2: “Berinert® has brought me a great improvement in my quality of life. Today, I feel more confident, more active, and freer.”

## Data Availability

The raw data supporting the conclusions of this article will be made available by the authors, without undue reservation.
